# Increased health and well-being in preschools (DAGIS): rationale and design for a randomized controlled trial

**DOI:** 10.1186/s12889-015-1744-z

**Published:** 2015-04-18

**Authors:** Suvi Määttä, Reetta Lehto, Mari Nislin, Carola Ray, Maijaliisa Erkkola, Nina Sajaniemi, Eva Roos, on the behalf of the DAGIS research group

**Affiliations:** Folkhälsan Research Center, Topeliuksenkatu 20, 00250 Helsinki, Finland; Department of Public Health, University of Helsinki, Helsinki, Finland; Department of Food and Environmental Sciences, University of Helsinki, Helsinki, Finland; Department of Teacher Education, University of Helsinki, Helsinki, Finland

**Keywords:** Health behavior, Preschool, Family, Stress, Socioecological model

## Abstract

**Background:**

Effective interventions that target socioeconomic status (SES) differences to avoid the potential widening of inequalities in health are needed. Children at preschool age is a valuable intervention target since sedentary behaviors, physical activity (PA), dietary behaviors, and sleep habits, jointly called the energy balance-related behaviors (EBRBs), are established in early childhood and tend to persist later in life. The interventions are most effective, when they focus on evidence-based factors. One potential factor associated with EBRBs and SES is children’s stress regulation, which receives special attention in this study. Based on the socioecological approach, the combinations of multiple levels (e.g. individual, environmental, societal) of analysis and diverse methodologies (e.g. surveys, observations, biological measurements) are used to assess the healthfulness of environments (e.g. social, physical, learning, policy) in preschool and family settings. The intervention aimed to diminish SES differences in EBRBs is then conducted in the preschool setting.

**Methods/design:**

The DAGIS study is divided into two phases. The first phase comprises focus group interviews and a cross-sectional survey. Parents and preschool personnel in low SES neighborhoods participated in interviews about children’s sedentary behaviors, dietary behaviors, and PA in 2014. In the cross-sectional survey beginning in autumn 2015, preschools will be recruited from a random sample of preschools in 3–5 municipalities in Southern Finland. A total of 800 children will wear an accelerometer for seven days. Children’s hair and saliva samples will be taken. Parents and preschool personnel will complete questionnaires on EBRBs, social and physical environments and SES factors. The quality of preschool environment is also observed. In the second phase, an intervention targeting to narrowing SES differences in EBRBs is conducted. The effects of the intervention will be evaluated in randomised controlled trial. The implementation of the intervention will also be evaluated.

**Conclusion:**

If effective, this unique preschool-based study will be able to narrow the SES differences in preschool children’s EBRBs. This study is anticipated to identify the most important modifiable factors in preschool and family environmental settings associated with children’s EBRBs, especially in children from low SES backgrounds.

**Trial registration:**

ISRCTN57165350 (January, 8th, 2015).

## Background

The prevalence of childhood obesity is a major public health concern, especially in groups of lower socioeconomic status (SES) [1]. Innovative, effective interventions that target to narrow SES differences are essential to avoid the potential widening of inequalities in health. The focus of obesity prevention and narrowing of SES differences should be on early childhood, when sedentary behavior, physical activity (PA), dietary behaviors, and sleep habits, jointly called energy balance-related behaviors (EBRBs), are established [2-4]. The EBRB habits formed in early childhood tend to persist throughout the life course [5]. Most 3-to-6-year-old children (e.g. about 80% of five-year olds in Finland [6]) from all SES backgrounds in Finland attend preschool, making preschool an ideal setting for interventions. Despite the need, interventions in preschool settings have been conducted less frequently than in school settings [7-9].

According to socioecological models [10,11], EBRBs are influenced by factors at multiple levels, including individual (e.g. age), environmental setting (e.g. preschool), and societal (e.g. socioeconomic neighborhood) [11]. Socioecological models propose that each environmental setting contains multiple types of environments that interact within each other [12]. In the preschool setting, for example, several environments (e.g. social, physical, policy, and learning) are known to have an influence on children’s EBRBs. Another key principle of socioecological models is the interaction across levels; for instance how societal level factors may moderate the association between environmental setting and behavior [12,13]. It is unclear whether the SES of the neighborhood in which a preschool is situated plays a role in the effect of the intervention. Research at multiple levels also enables identification of new potential *modifiable factors* for use in intervention. One potentially modifiable factor is children’s stress regulation. Recent findings have concluded that stress may be a possible direct predictor of obesity in children as well as an indirect predictor through unhealthy EBRBs [14]. Stress also seems to be associated with SES and EBRBs [14]. However, it is unclear how the preschool setting is associated with children’s stress regulation. To summarize, recognizing the evidence-based modifiable factors, especially in the low SES groups of children, and creating an intervention focusing on these factors, might be beneficial for improving health and simultaneously narrowing health inequalities.

Combinations of multiple levels of analysis and diverse methodologies (e.g. surveys, observations, biological measurements) are valuable for assessing the healthfulness of several environments within a certain environmental setting [15]. Therefore, a comprehensive needs assessment will more likely lead to effective behavioral changes because it concentrates on theory-driven, evidence-based factors across multiple levels and multiple environments in an intervention [13]. An interesting question is whether *an intervention focused on multiple environments in preschool and family settings can balance children’s EBRBs and also diminish SES differences in EBRBs.* Answering this question is the main aim of the DAGIS (Increased health and wellbeing in preschools) study. The purpose here is to describe the design, theoretical framework, recruitment process, and methodology of the DAGIS study.

## Methods/design

### Study design and aims

**Figure 1:**
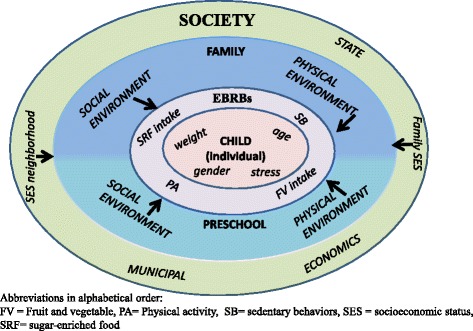
The theoretical model of the DAGIS study.

The DAGIS study is based on a socioecological framework aimed at evaluating the factors associated with preschoolers’ EBRBs within and across several levels (Figure 1). The general objective of the study is to reduce socioeconomic inequalities in children’s EBRBs and promote a healthy lifestyle among Finnish preschool children. The preschool and family serve as environmental settings with several environments (e.g. social, physical, learning) that are explored. The focus of EBRBs in this study will be on fruit and vegetable intake, sugar-enriched food intake, PA and sedentary behaviors.

To achieve this aim, a stepwise approach is implemented and specific objectives are set as follows:

1. To perform a thorough analysis of the most important behaviors contributing to children’s energy balance and their most significant modifiable factors with a special focus on the low socioeconomic backgrounds (years 2014–2016, referred to as phase 1 (P1) later in this article),
2. To identify the mediators and moderators of EBRBs in different sub-populations based on age, gender, and SES background (years 2014–2016, P1),
3. To design a preschool-based, family-involved intervention (years 2014–2016, P1),
4. To implement a preschool-based, family-involved intervention (years 2017–2019, referred to as phase 2 (P2) later in this article),
5. To test the implementation and effectiveness of the intervention in a randomized control trial (RCT) (years 2017–2019, P2).

**Figure 2:**
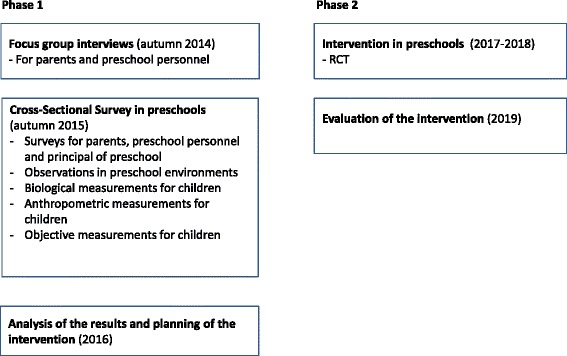
The study phases and timetable.

Aims 1–3 are carried out in P1 (Figure 2). P1 includes both focus group interviews and a cross-sectional survey. Several environments (e.g. social, physical, policy and learning) in preschool and family environmental settings are investigated using diverse methodologies (e.g. interviews, surveys, observations, biological measurements).

P2 comprises an intervention with a randomized controlled trial (RCT) in the preschool setting (Figure 2). In P2, the intervention is carried out based on the results of P1, previous research, and behavioral change theories [11,16]. The implementation of the intervention is evaluated throughout methods following the RE-AIM framework [17].

### Study context

In Finland, all the children under school-age have a subjective right to a place in preschool. The municipalities are responsible for arranging the preschool services, for their quality and supervision. Preschool groups are usually divided according to age into under 3-year-olds and 3-6-year-olds. In addition, preschools may also provide compulsory, but free-of-charge pre-primary education for children in the year preceding the start of their compulsory education. The school begins at the age of 7 in Finland. The majority of children in preschool attend to full-time day care, but part-time care and round-the-clock care are also provided [18,19]. In the DAGIS-study, the focus will be on the preschool groups over 3-year-old children, but the pre-primary education groups are not included.

Children in preschool receive the necessary meals depending on the length of their day, e.g. breakfast, lunch and a light afternoon snack for children in full-time daytime care. National dietary guidelines exist for young children including also recommendations for meals served in preschools [20]. Meals are included in the monthly fee. Families pay fees that in full-day provision vary between 0-283€ (as of 2014) depending on the size and income of the family. The fee is determined by the Act [18,19].

The number of personnel according to legislation is that there may be a maximum of seven 3-6-year-old children in full-time care to each early childhood professional. The qualification for the leader of each preschool group is a university or university of applied science degree and all preschool personnel must have at least an upper secondary-level qualification in social welfare and health care (ISCED 3). Each municipality has its own educational plan for preschools, but the practices followed are mainly decided upon the principal of each preschool. The variation in practices and policies can therefore be wide and is dependent on both the municipality and the preschool [18,19].

### The ethics statement

The Ethics approval for the focus group interviews was obtained on May 15th, 2014, from the Coordinating Ethics Committee of the Hospital District of Helsinki and Uusimaa. The Ethics approval for the cross-sectional survey was obtained on February 24th, 2015, from the University of Helsinki Review Board in the humanities and social and behavioral sciences. In each part of the study, an informed consent will be delivered to principal of preschool and child’s guardian. Consent for the children to participate in the study will be signed by one legal guardian, referred as parent(s) hereafter. Participants have the right to withdraw from the study at any time without consequences. The study is conducted according to the Declaration of Helsinki and good scientific practices. The DAGIS project follows the CONSORT guidelines for Randomized Controlled Trials [21].

### Participant recruitment

The participating municipalities in this study are from Southern Finland. The selections of the municipalities are based on SES indicators (larger variation of educational level, income level, and higher Gini coefficient) according the national statistics [22].

The preschools for focus group interviews were recruited in autumn 2014. Focus group interviews were conducted separately for parents and preschool personnel in low SES neighborhoods. A low SES neighborhood was defined as belonging to the lowest tertiaries of educational and income level in the municipality according to municipality statistics. Parents participating in interviews needed to have at least one 3-5-year-old child in the preschool situated in a low SES neighborhood, and preschool personnel needed to work in a preschool situated in a low SES neighborhood.

Three to five municipals for the cross-sectional survey will be recruited in spring 2015. The preschools for the cross-sectional survey will be recruited from a random sample of preschools. The recruitment focuses on preschool groups over 3 years or older children. After a preschool consents to participate, the parents that have a child over 3 years old in the preschool will be contacted, and their willingness to participate in the study is asked. An inclusion criterion for the preschool group will be that at least 30% of the children in the group participate. In the consent form, the educational background and the current work status of the parent and possible other partner will be asked. Based on this background information, the research group controls the participation rate all the time, and possible new preschool recruitments are done to achieve a wide variation of SES background among the participants.

In P2, half of the recruited preschools will be randomized to an intervention group and the other half to a control group. Participant recruitment for P2 follows otherwise the previously presented guidelines.

#### Sample size and power calculations

Power and sample size calculations were conducted for sedentary behaviors, fruit intake, vegetable intake and sugar-enriched food intake separately. The sample size varied in these calculations from 600 to 800 children. As the required sample size was largest for estimating the intake of fruit, the power and sample size calculations are based on its results in P1. Power calculations were done to ensure that the number of participants is sufficient to detect defined differences between groups. Based on previous studies [23], the mean intake of fruit among 4-year-old children in Finland is 101 g/day, and the standard deviation (SD) is 82 g [24]. To detect a 20-gram difference between two groups with 80% power and a significance level of 0.05, 265 children per group are needed. We want to be able to compare the highest and lowest tertiaries of SES. To detect a 20-g difference between the lowest and highest tertiaries, and therefore we need a sample of 795 children, rounded up to 800 children. With an average preschool group size of 20 children and the assumption that in average 60% of children (n = 12 per group) would participate in the survey, a total number of 67 preschool groups is required to obtain a sufficient children in P1.

The targeted modifiable factors in the intervention will be specified based on results from P1 and the power and sample calculations will therefore be re-conducted in the year 2017 to assure the effectiveness of intervention.

### Measurements and protocol

**Figure 3:**
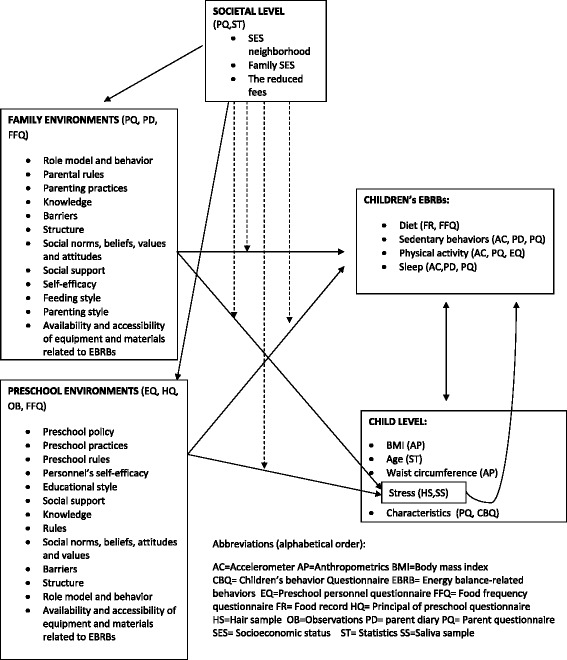
The proposed associations of preschool and family environmental settings with children’s energy-balance related behaviors.

Assessments for the DAGIS study are based within and across the spheres of the socioecological model presented in Figure 3.

#### Focus group interviews

Semi-structured interviews including similar themes for each interview was carried out for parents and preschool personnel, with 2–6 participants in each interview. The research team developed, pre-tested, and refined the semi-structured questioning route separately for parents and preschool personnel. The questioning route was based on the socioecological framework, including both individual and environmental factors related to children’s PA, sedentary behaviors and dietary behaviors either in the family or at preschool.

A moderator led the discussion to ensure that all the themes were discussed, and a co-moderator made notes during the discussion. At the beginning of the interview, participants completed a questionnaire that included SES factors and warm-up questions related to the themes. During the interview, the participants received healthy refreshments (e.g. fruits and water), and a taste school of fruits and vegetables with guided play session was organized for the children of participants. After the interview, each participant received a small recompense of participation. The moderator and the co-moderator discussed their impressions, also covering group characteristics, and identified issues that affected the interviews in a debriefing session held immediately after the interviews. The focus groups were audio recorded and transcribed verbatim.

#### Cross-sectional survey with needs assessment

The second data collection phase of P1 will begin in the year 2015. Children, their parents, and preschool personnel will participate in this phase.

##### Child level

To measure children’s diet, several *dietary assessment methods* are used. A pre-coded *food record* is a reliable method for estimating food intake. The record is filled in on two preschool days and one weekend day separately by parents and preschool personnel. A picture booklet will be developed and validated to aid the estimation of portion sizes of commonly eaten foods in preschools and at home. Accordingly, foods and drinks eaten at preschool will be photographed before and after each meal by the research personnel. Example portions with weighed amounts will be photographed to be used as an aid in calculation. An easy-to-conduct children’s food frequency questionnaire (FFQ) will be developed and validated. The FFQ will measure a longer-term habitual food intake (7 days) comprising of 46 food items and six additional questions. The FFQ is filled by parents. All of these assessments will be developed and pre-tested in 2014 and 2015.

To measure children’s sedentary time and PA, children wear an *accelerometer* (Actigraph wGT3X-BT, ActiGraph, Pensacola, FL) for one week. Previous studies have indicated that one week is adequate for children to determine habitual PA and sedentary time [25]. Simultaneously with accelerometer use, parents are asked to report preschool hours and sleeping times of their children in a *diary*. In the same diary, the parents also report children’s sedentary behaviors daily. The modified diary based on a validated diary [26] is used for reporting sedentary behaviors. The research group will also collect information about the weather each week, which is taken into account in the analyses of the accelerometer data.

To measure stress regulation, *saliva and hair samples* are taken. Stress is estimated from cortisol and alpha-amylase samples from saliva. Measuring cortisol levels from saliva samples is a well-established and non-invasive method to examine functioning of a child’s hypothalamic–pituitary–adrenal axis. Combining alpha-amylase measurement (from the same saliva samples), an indicator of sympathetic-adrenal-medullary axis functioning, with the cortisol measurement provides a more complete picture of stress regulation [27]. We have previously demonstrated that we are able to conduct data collection, and measure reliably children’s stress regulation at the preschool and at family settings [28-31]. Further, we will utilize the data collection procedure used in the study by Vermeer et al. [32] to study more precisely the variation in the salivary cortisol and alpha-amylase levels during the preschool day [32]. By combining data collected at home and at the preschool, we will obtain baseline data for detecting the diurnal variation, and data that describes children’s stress reactivity at the preschool.

In this study, the salivary cortisol and alpha-amylase samples will be collected over the period of two days—one weekend day and one day care day. For the purposes of detecting the diurnal secretion of hormones, in total five samples will be collected with the help of parents during one weekend day at home. The first sample collection will be conducted as follows: (1) on the awakening (2) half an hour after awakening, (3) an hour after awakening, (4) before lunch (5) just before bedtime. The second collection will be organized during the day at the preschool, and early childhood professionals will take the samples. The sampling times will be following: (1) before lunch, (2) between 14:00 and 15:00, (after the naptime, but before afternoon snack.) Chronic stress level of children is estimated by cortisol in hair samples, which reflects long-term cortisol secretion [33]. A hair sample of 5–20 g will be cut close to the scalp.

Children’s height, weight, and waist circumference will be measured by the research group at the preschool.

##### Family environments

Parents will complete a questionnaire about the family status, SES, child characteristics, FFQ of own food intake (both parents separately), and family environments. Family status includes e.g. size of family and marital status. SES includes e.g. educational level, household income level, and occupational status of the parents. The section of child characteristics investigates the child’s reactivity to different daily situations. Child characteristics are measured by the very short form of the Children’s Behavior Questionnaire [34]. The parents’ habitual food consumption (7 days) will be measured by FFQ (48 items + 7 additional questions).

Family environments are defined as the social and physical environments in the family setting. In this study, the social environment of the family is defined as practices, social norms and beliefs, parenting practices, self-efficacy, encouragement, parenting style, barriers, and structures in interaction between parent and child. The section of physical environment includes questions on availability and accessibility of a physical environment related to children’s EBRBs. The questionnaire is under development and will be based on previously validated questions [35-37]. The questionnaire will be pre-tested in the Finnish context before use both in autumn 2014 and in spring 2015. All the material for parents will be provided in Finnish, English and Swedish.

##### Preschool environments

*Observations and questionnaires* will be used to explore the environments of preschools.

The preschool environments will also be observed by using the previously developed *observation* methods. The observation protocols were pre-tested and modified into Finnish context in autumn 2014. The physical environment of the preschool is defined in this study as indoor and outdoor facilities related to children’s EBRBs (e.g. availability of electronic equipment, structure of yard, PA facilities, and meal-time structure). The physical environment will be assessed by previously used observation protocols – the combinations of the Environment Policy Assessment and Observation (EPAO) [38] and the Outdoor Play Environment Categories (OPEC) [39]. The observation protocol includes also a short questionnaire for the leader of observed preschool group. The short questionnaire includes items that cannot be observed directly, but are related to physical environment (e.g. weekly structure of the preschool group).

Observations on the learning environment focus on quality aspects of the learning environment and adults’ engagement in preschools. The preschool personnel and their activities will be observed by using the Learning Environment Assessment (LEANS) [40]. The focus of this evaluation includes classroom arrangement, schedules and transitions, classroom activities, team planning, and behavior plans [40]. Teacher’s pedagogical sensitivity in interaction with children will be analyzed using the Adult Engagement Scale (AES) [41].

Preschool personnel will answer a *questionnaire* covering social and physical environment in preschools. Social environment in preschool is defined as practices, social norms and beliefs, encouragement, pedagogical efficacy, pedagogical style, barriers and structures in interaction between early educator and child in the preschool setting. The section of physical environment includes questions on accessibility and availability of the physical environment related to children’s EBRBs. In addition, preschool personnel’s FFQ of own food intake (items 48) is a component of the questionnaire for preschool personnel; this FFQ is being developed during this study.

A *questionnaire* will be created for preschool principals. This questionnaire will focus on the policy environment of the preschool, defined as written policies and practices related to EBRBs (e.g. frequency of PA lessons, birthday protocols).

Catering personnel answer a *questionnaire* focusing on meal preparation and catering personnel’s practices and attitudes related on preschool meal preparation. It also enquires personnel’s knowledge and opinions on policies and practices related to the quality and quantity of served food, and opinions on overall co-operation between catering personnel and preschool personnel.

All the questionnaires are under development and will be pre-tested in the Finnish context before use both in autumn 2014 and in spring 2015.

##### Societal level

To measure the SES neighborhood, the preschools will be ranked on the basis of educational and income level statistics by the neighborhoods in their municipalities. The municipal registers will also provide information about the reduced fees in each preschool, and therefore the preschools will also be ranked by proportion of children getting reduced fee.

#### Intervention with RCT

##### The measurements in RCT

The baseline measurements will be conducted in autumn 2017 and the follow-up measurements in spring 2018 directly after the six-month intervention ends. The baseline and follow-up measurements for children, parents, and preschool personnel will be a shortened version of those in P1. The measurements in the RCT will focus on the EBRBs of the children that the intervention aims to improve and on the modifiable environmental factors that the RCT will intervene. Knowledge and experiences of national and international collaborators will be taken into account in the planning the measurements in the RCT.

##### Content of the intervention

The intervention focusing on several environments at preschool and at family settings will be conducted. The main focus in the intervention will be on several modifiable environmental factors at preschool setting, but an intervention component for family setting also will be conducted through preschool. The content of the intervention will be based on the results from the P1. After the P1, we will firstly have determined the children’s EBRBs with the largest SES differences to be focused on this intervention. Secondly, we will know the most significant modifiable environmental factors that are associated with the SES differences in these EBRBs in both preschool and family settings. By intervening on these modifiable factors, the general objective of the study will be reached; to reduce socioeconomic inequalities in children’s EBRBs and promote a healthy lifestyle among Finnish preschool children.

The intervention will take the empowerment approach - that is the preschool personnel and parents will be actively involved in all the stages of the intervention planning and implementation. The user-friendly methods used in this intervention will however be as standardized as possible in all preschools. Several different kinds of methods (e.g. seminars, guide books, daily practices) will be used.

##### Implementation of the intervention

The implementation of the intervention will follow the RE-AIM framework [17,42]. The dose delivered (intervention components), completeness of intervention components, and fidelity of implementation will be recorded by qualitative (site visits) and quantitative (logbooks, checklists) methods. One to three researchers will make a few site visits to the preschool during the intervention phase. Checklists and logbooks assess the preschool personnel’s reports of intervention completeness, fidelity measures, possible barriers to implementation, and children’s responsiveness to the components in intervention. In addition, preschool personnel’s satisfaction with and attitudes towards training and support activities and intervention components will be assessed. The process evaluation also includes assessment of the preschool practices, contextual elements, and preschool characteristics related to PA, sedentary behaviors and dietary behaviors. The follow-up survey for the preschool personnel and the parents in the control groups will also include questions about their awareness of the DAGIS intervention.

#### Data analysis

The statistical programs The Statistical Package for Social Sciences (SPSS) (IBM Inc, Chicago, IL, USA) and Mplus (Muthen & Muthen, USA) and the qualitative data analysis software Nvivo 10 (QSR international Pty Ltd., Doncaster, Victoria, Australia) will be used to analyze the quantitative data. The focus group interviews are analyzed with the help of Nvivo 10. A data framework to code the data, which is based on the major themes of the questioning route, is used. Pre-coded food records will be entered and processed with a software program AivoDiet that uses the Fineli Food Composition Database. Fineli was developed, and is being continuously updated, by the Finnish National Institute for Health and Welfare [43].

In the cross-sectional survey, several mediation analyses will be conducted to determine the most influential mediators in the associations of EBRBs and preschool and family environments. Moderating effects by subgroups will be explored by testing for interactions in regression and logistic regression models. A clustering effect by preschools will be taken into account with multilevel analyses. The effect evaluation of the intervention will be based on multilevel analyses between the intervention and control schools on follow-up values adjusted with baseline values. The moderating effect of SES backgrounds (neighborhood and family SES) will be tested. Sub-group analyses will be conducted to determine whether certain interventional strategies are superior for a particular sub-group of SES neighborhoods.

## Discussion

The DAGIS study is anticipated to shed light on effective methods for obesity intervention in early childhood. The study utilizes a socioecological model that allows exploration of relationships among EBRBs within and across multiple environments. Examining EBRBs in multiple environments can provide insights into the most significant modifiable factors in different environments in both preschool and family settings. In addition, we hope to determine how the stress is associated within and across multiple environments. An intervention can provide substantial public health benefits through healthier EBRBs and diminished SES differences in EBRBs.

A key strength of this study is a design embedded within a socioecological approach that combines multiple levels of analysis and diverse methodologies for assessing the healthfulness of different environments. Preschool environments combined with family environments provide promising settings for balancing EBRBs, especially when multilevel strategies are applied. A further strength is the recruitment strategy that will take into account SES backgrounds in each stage. When randomization is achieved, the results will provide insights into the most modifiable factors associated with children’s EBRBs at different SES groups. This study is one of few to extensively conduct process evaluation to provide thorough documentation of the implementation of the intervention. The process evaluation is based on several methods accurately assess the effects of the intervention.

A potential weakness of the study is that the measures of family and preschool habits are mainly based on reports from parents and preschool personnel and not on objective evaluation by the researchers. However, the researchers do make observations in the preschool environment. Although the cross-sectional survey aims to uncover all the potentially modifiable factors in the family and preschool environments, the number of questions that are included in the questionnaires will probably be restricted since willingness to participate is lowered if the questionnaires are too burdensome. Further potential challenge is that the recruitment aim of variety of SES backgrounds may not be achieved; especially low SES families might be more challenging to recruit. The research group has therefore developed plans to motivate participation by providing recompense of participation.

As most health habits are established in early childhood, we believe that an intervention at multiple environments in preschool and family settings can balance children’s EBRBs and diminish SES differences in EBRBs.

## Abbreviations

PA: Physical activity
SES: Socioeconomic status
EBRBs: Energy balance-related behaviors
SD: Standard deviation
EPAO: Environment Policy Assessment and Observation
OPEC: Outdoor Play Environment Categories
LEANS: Learning Environment Assessment
AES: Adult Engagement Scale
FFQ: Food frequency questionnaire
RCT: Randomized controlled trial
P1: Phase 1
P2: Phase 2
